# Efficacy of Transcranial Magnetic Stimulation (TMS) on Negative and Cognitive Symptoms in Schizophrenia – a Systematic Review and Meta-Analysis

**DOI:** 10.1192/bjo.2024.237

**Published:** 2024-08-01

**Authors:** Roopa Rudrappa, Andrea Cavanna, Hugh Rickards, Mohammad Zia Ul Haq Katshu

**Affiliations:** 1 Derbyshire Healthcare NHS Foundation Trust, Derby, United Kingdom; 2 University of Birmingham, Birmingham, United Kingdom; 3 Birmingham and Solihull Mental Health NHS Foundation Trust, Birmingham, United Kingdom; 4 Institute of Mental Health, School of Medicine, University of Nottingham, Nottingham, United Kingdom; 5 Nottinghamshire Healthcare NHS Foundation Trust, Nottingham, United Kingdom

## Abstract

**Aims:**

Traditional antipsychotic treatment improves positive symptoms in schizophrenia but has little impact on negative and cognitive symptoms. TMS is a non-invasive neuromodulation technique which has been suggested to impact negative and cognitive symptoms of schizophrenia. This systematic review critically appraised the research evaluating the effect of TMS on negative and cognitive symptoms of schizophrenia. Furthermore, we carried out a meta-analysis of randomised controlled trials of the effect of TMS on negative symptoms in schizophrenia.

**Methods:**

Systematic review was carried out according to PRISMA guidelines. Cochrane Library, Ovid Medline, Science Direct and PubMed databases were searched for relevant studies using the search terms: “transcranial magnetic stimulation” OR “TMS” OR “repetitive transcranial magnetic stimulation” OR “r-TMS” OR “theta burst stimulation” OR “TBS” AND “negative symptoms” OR “cognitive dysfunction” OR “cognitive impairment” AND “schizophrenia” OR “psychosis”. Only randomised controlled trials evaluating the effect of TMS (rTMS or iTBS, intermittent theta burst) on negative and/or cognitive symptoms in schizophrenia were selected. Thirty-three studies were included in the systematic review. The Standardised mean difference (SMD) with 95% confidence interval (CI) was calculated for each study and pooled across studies using an inverse variance random effect model.

**Results:**

Sixteen studies demonstrated significant improvement in negative symptoms with a superior effect of TMS compared with sham intervention. Eight studies showed improvement in certain domains of cognition and one study showed a delayed effect on negative symptoms. Studies which showed positive effects on negative symptoms have used similar TMS parameters such as 10 Hz over L-DLPFC (Left dorsolateral prefrontal cortex) except for a few studies. Ten studies reported negative results for negative and/or cognitive symptoms, TMS parameters and duration of treatment used varied among these studies. Overall, SMD for SANS (Scale for Assessment of Negative Symptoms) was 0.89 (95%CI: 0.46–1.32, P < 0.00001) and for PANSS-N (Positive and Negative Syndrome scale-negative) was 0.67 (95%CI: 0.22–1.12, P < 0.00001), both in favour of TMS. The heterogeneity of the included studies was high, I^2^- 85% for SANS and 92% for the PANSS-N subscale with a small to moderate risk of publication bias.

**Conclusion:**

High-frequency rTMS is more effective than sham in improving negative and cognitive symptoms in schizophrenia. Our results suggest the need for well-designed randomised controlled trials with larger sample sizes and standard harmonised cognitive assessments to assess the effect of TMS on negative and cognitive symptoms to provide sufficient evidence for inclusion in routine clinical practice.

